# Study on High-Velocity Impact Perforation Performance of CFRP Laminates for Rail Vehicles: Experiment and Simulation

**DOI:** 10.3390/biomimetics8080568

**Published:** 2023-11-27

**Authors:** Xuanzhen Chen, Yong Peng, Kui Wang, Xin Wang, Zhixiang Liu, Zhiqiang Huang, Honghao Zhang

**Affiliations:** 1 Key Laboratory of Traffic Safety on Track of Ministry of Education, School of Traffic and Transportation Engineering, Central South University, Changsha 410075, China; 2 Joint International Research Laboratory of Key Technology for Rail Traffic Safety, Central South University, Changsha 410075, China; 3 Key Laboratory of High Efficiency and Clean Mechanical Manufacture (Ministry of Education), School of Mechanical Engineering, Shandong University, Jinan 250061, China

**Keywords:** high-velocity impact, CFRP laminate, finite element method, failure

## Abstract

To study the perforation performance of CFRP laminates for rail vehicles under high-velocity impact from foreign objects, impact tests on CFRP laminates at a velocity of 163 m/s were carried out, and a corresponding finite element model was established using ABAQUS and verified. The user-defined material subroutine combined the material strain rate hardening effect and the 3D-Hashin damage criterion. The effects of impact velocity, impact object shape, and oblique angle on the perforation performance of CFRP laminates are discussed. Results show that impact velocity positively correlates with impact peak force and residual velocity. Laminates can be perforated by projectiles with a velocity above 120 m/s, and impact velocity greatly influences delamination below 140 m/s. Three shapes of projectile impacting laminates are considered: spherical, cylindrical, and conical. The conical projectile penetrates the laminate most easily, with the largest delamination area. The cylindrical projectile with a flat end suffers the most resistance, and the delaminated area is between the impact conditions of the conical and spherical projectiles. Increasing the angle of inclination increases the impacted area of the laminate and the extent of damage, thus dissipating more energy. The projectile fails to penetrate the laminate when the oblique angle reaches 60°. CFRP composite structures penetrated by high-speed impacts pose a significant threat to the safety of train operations, providing an opportunity for the application of bio-inspired composite structures.

## 1. Introduction

Using lightweight car bodies is essential for high-speed trains to reduce energy consumption and greenhouse gas emissions [1,2]. Compared with traditional rail vehicle body materials such as stainless steel and aluminum alloy, CFRP has become the most promising material candidate in the future due to its high specific strength, high modulus, and weather-shield durability. There have been several successful cases of CFRP structure application in the field of rail transit, such as Japan’s E4 driver’s cab, N700 series car roof, French TGV Duplex train body [3], and South Korea’s Title Train eXpress [4]. Recently, CRRC Corp. Ltd., China’s largest rail transportation equipment maker, unveiled a maglev train capable of 600 km/h whose body is made of carbon fiber-reinforced composite materials [5]. At present, researchers have carried out research on static performance [6,7,8,9], fire safety [10], low-speed impact resistance of foreign objects [11,12], fatigue life [13], and natural frequency [14] of composite train bodies or bogies. With the increasing speed of trains, the risk of high-speed train bodies being impacted by external objects increases, making it necessary to carry out research on the impact resistance of CFRP rail vehicle bodies.

The parameters affecting the performance of fiber-reinforced composites under dynamic impact can be classified into four types, including material-based parameters, geometry-based parameters, impactor-based parameters, and environmental-based parameters [15]. Many studies have also carried out parametric response studies for high-velocity impacts through experiments and simulations [16,17,18,19,20,21,22,23,24,25,26,27,28,29,30]. The mechanical properties of the laminate itself have a decisive influence on the resistance to high-velocity impact. The thickness of the plate affects the failure mode. The experimental study by Fujii et al. [19] showed that the quasi-static strength and failure strain of fibers have no effect on the failure mode of the thin plate or the front layers of the thick plate but have a more significant impact on the failure mode of the back of the thick plate. The shear strength of the matrix is a significant influence. Karthikeyan et al. [20] investigated the effect of matrix shear strength on the dynamic response. The CFRP plate with low shear strength is penetrated progressively. The one of high matrix shear strength fails by a combination of cone-crack formation and comminution of plies. The ballistic limit of fiber composite plates increases with decreasing matrix shear strength. Impact velocity is considered to be positively related to the area of the damaged area [21,22,23]. An analytical model was developed by Lopez-Puente et al. [24] to predict the residual velocity as well as the ballistic limit. Another critical factor is the obliquity of HVI [25,26,27,28]. Yuan et al. [23] investigated the laws governing the effects of projectile size and mass on the impact response of CFRP laminates. They found that at high-energy impacts, large-sized projectiles imparted a large amount of delamination to the plywood accompanied by fiber breakage and peeling, while small-sized projectiles imparted shear clogging and limited delamination. The oblique impact increases the projectile penetration path length, increasing the ballistic limit velocity. The increased damaged area after penetration leads to more energy dissipation [24]. However, below the ballistic velocity limit, the damage caused by the oblique impact is less than that caused by the normal impact [27]. Xie et al. [28] established an energy dissipation prediction model linking energy dissipation with ballistic limit and impact angles. The impact resistance of the laminate changes when the external environment changes [29]. The experiments performed by Liu et al. [30] showed that hygrothermal conditions improve the impact resistance of the CFRP laminate and that the laminate is more prone to failure in low-temperature environments. The researchers also considered the impact response at different impact positions, and the impact position was considered to be an essential factor affecting the impact response as well [31,32]. Impacts produced different damage patterns in the center and non-center locations, with delamination being more pronounced in the former than in the latter [31]. The damage tolerance at the center of the laminate is higher than at the edges [32].

The mechanical properties of materials at high strain rates are significantly different from those at low strain rates [33,34]. The HVI of CFRP laminates is a typical high-strain-rate condition. CFRP is a strain-rate-sensitive material [35,36,37,38,39,40]. Its tensile strength at a high strain rate is significantly higher than that at a low strain rate, especially when the strain rate is greater than 50 s^−1^ [39]. However, published studies rarely consider the strain-rate effect of the material in HVI simulations for CFRP laminates. The strain-rate effect dramatically impacts the results in HVI simulations and should be considered an important material property.

This study carried out the HVI experiments and simulations of CFRP laminates for rail vehicles. The ballistic performance of CFRP laminates at a projectile velocity of 163 m/s was tested using an aerodynamic high-speed impact test apparatus. A high-speed impact finite element model was established based on ABAQUS/Explicit. The user materials subroutine in the model integrates the 3D-Hashin failure model and strain rate effects. The test high-speed photography’s velocity history and deformation process verify the model’s validity. Finally, the effects of projectile velocity, single-head shape, and incident angle on impact damage are discussed.

## 2. Materials and Methods

### 2.1. Specimen and Experimental Conditions

The CFRP laminates consist of 14 plies of unidirectional T800/epoxy resin prepregs. An automatic layup machine lays the prepreg, and the CFRP laminate is thermoformed and cured at 1 Mpa of pressure and 180 °C for two hours. Finally, it is machined into specimens by mechanical processing. The stack sequence of composite is ${\left[\pm 45{}^{\circ}\right]}_{7s}$. The CFRP prepreg has a nominal ply with a thickness of 0.15 mm. The dimensions of the tested laminate is 200 mm×200 mm×4.2 mm. A stainless-steel ball with a diameter of 20 mm is used for the high-speed impact test projectile and its mass is 33 g.

**Figure 1:**
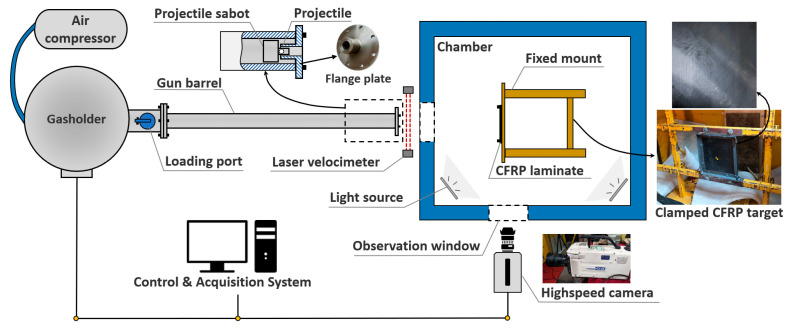
Schematic diagram of the high-velocity ballistic test system.

**Figure 2:**
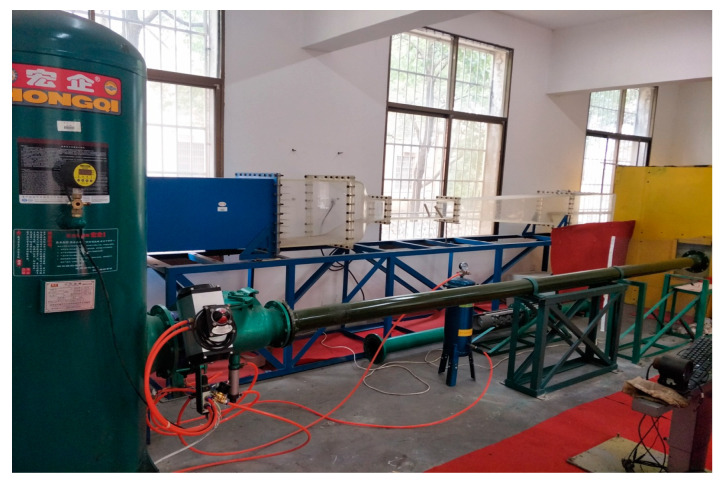
Photo of the experimental setup.

The experimental device used in this study is the high-velocity ballistic test system of the Key Laboratory of Traffic Safety on Track, Ministry of Education of Central South University. Figure 1 shows the schematic diagram of the experimental setup. Figure 2 is a photo of the experimental setup.

**Figure 3:**
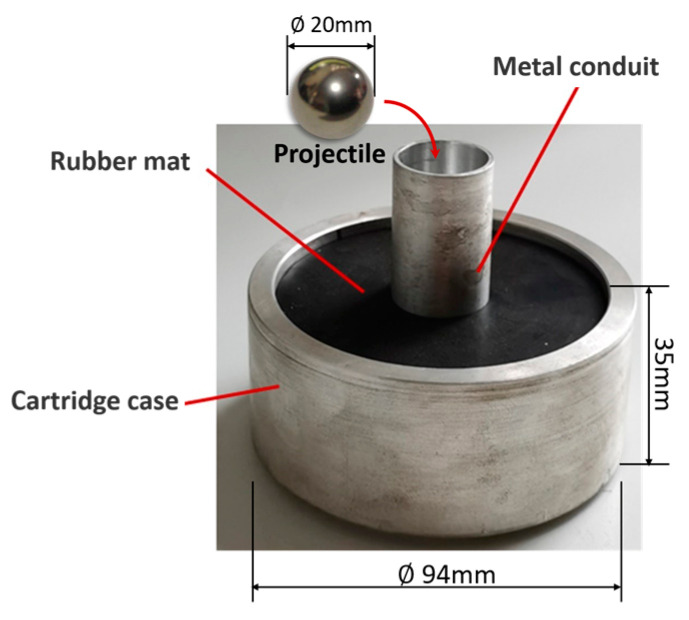
The projectile and sabot.

The test system consists of a one-stage gas gun launcher, a velocimeter, a specimen fixture, a high-speed camera, and a control and acquisition system. A gasholder with a volume of 0.6 $m^{3}$ can be pressurized by the air compressor to provide launch energy. A double infrared sky screen velocimeter is placed next to the exit of the barrel to record the shooting speed of the projectile. The CFRP laminate sample is fixed in an enclosed chamber. Both the gasholder valve and the high-speed camera are computer-controlled. An aluminum sabot with a conduit adapts to the barrel diameter. It is filled with a rubber mat for cushioning. The sabot and its main dimensions are shown in Figure 3. The sabot preloaded with the projectile is put into the rear of the barrel from the loading port, and the stock will be pushed along the barrel by the high-pressure gas until the flange blocks it at the outlet of the barrel. Due to inertia, the projectile is separated from the sabot, flying out of the barrel, and shooting towards the target in the chamber. The high-speed camera is perpendicular to the trajectory, and the window captures the impact process. The high-speed camera is NAC’s Memrecam HX-6E, which can capture images with a resolution of 1280 × 720 at a frame rate of 5000 fps. As the high-speed impact process lasts less than 1 ms, taking into account the size of the screen, the frame rate is 20,000 fps. The camera is automatically triggered by a switch installed at the end of the gun barrel, and the captured image data is transmitted to the computer.

### 2.2. Finite Element Simulation

A finite element analysis model of the experiment was established to analyze the HVI process of CFRP laminates in detail. Commercial software, ABAQUS/Explicit (version 6.13-4), is employed for simulation. The key to simulating the high-speed impact process of laminates is to reproduce the damage and failure of laminates accurately. The HVI failure process of laminates includes two damage modes: intra-laminar damage and delamination (inter-laminar damage).

#### 2.2.1. Intra-Laminar Damage Model

Intra-layer damage includes fiber tensile damage, fiber compression damage, matrix tensile damage, and matrix compression damage. 3D-Hashin criteria were adopted for fiber breakage and matrix crack, as shown in Table 1.

When the damage indicator $F_{j}^{i}$ is greater than one, the damage of the corresponding mode initiates, where i=t, c for tensile and compression, and j=f, m for fiber and matrix, respectively. $\sigma_{11}$, $\sigma_{22}$, $\sigma_{33}$, $\tau_{12}$, $\tau_{13}$, and $\tau_{23}$ are stress components. $S_{1t}$ and $S_{1c}$ are longitudinal tensile and compression strength. $S_{2t}$ and $S_{2c}$ are transversal tensile and compression strength. $S_{12}$, $S_{13},$ and $S_{23}$ are three shear strengths. The corresponding degradation constants $d_{ft}$, $d_{fc}$, $d_{mt}$, and $d_{mc}$ will be activated to degrade material properties when the damage initiates [41]. Material properties are provided by manufacturers and ref. [42], given in Table 2.

After material damage, the material’s stiffness is considered to have degraded to a certain extent. The stiffness matrix of a material can be derived from its stress-strain relationship. CFRP laminates can be treated as a transversely isotropic material. The stress—strain relation is given by:

(1) $$\varepsilon =S^{d}\sigma$$

where ε and σ are the strain vector and stress vector, respectively.

(2) $$\sigma ={\left[\begin{array}{cccccc} \sigma_{11} & \sigma_{22} & \sigma_{33} & \tau_{12} & \tau_{23} & \tau_{31} \end{array}\right]}^{T},\;\varepsilon ={\left[\begin{array}{cccccc} \varepsilon_{11} & \varepsilon_{22} & \varepsilon_{33} & \gamma_{12} & \gamma_{23} & \gamma_{31} \end{array}\right]}^{T}$$

$S^{d}$ denotes the degraded flexibility matrix expressed as:

(3) $$S^{d}=\left[\begin{array}{cccccc} \frac{1}{d_{f}E_{11}} & -\frac{\nu_{21}}{E_{22}} & -\frac{\nu_{31}}{E_{33}} & & & \\ -\frac{\nu_{12}}{E_{11}} & \frac{1}{d_{m}E_{22}} & -\frac{\nu_{32}}{E_{33}} & & & \\ -\frac{\nu_{13}}{E_{11}} & -\frac{\nu_{23}}{E_{22}} & \frac{1}{E_{33}} & & & \\ & & & \frac{1}{d_{f}d_{m}G_{12}} & & \\ & & & & \frac{1}{d_{f}d_{m}G_{23}} & \\ & & & & & \frac{1}{d_{f}d_{m}G_{31}} \end{array}\right]$$

where $E_{11}$, $E_{22},$ and $E_{33}$ represent Young’s moduli in three directions. $G_{12}$, $G_{23}$, and $G_{31}$ are three shear moduli in three planes. $\nu_{ij}\;(ij=12,\;21,\;13,\;31,\;23,\;32)$ represents Poisson’s ratio. The stiffness matrix can be deduced as:

(4) $$C^{d}=\frac{1}{\Delta }\left[\begin{array}{cccccc} d_{f}E_{11}(1-d_{m}\nu_{23}\nu_{32}) & d_{f}d_{m}E_{11}(\nu_{21}+\nu_{23}\nu_{31}) & d_{f}E_{11}(\nu_{31}+d_{m}\nu_{21}\nu_{23}) & & & \\ & d_{m}E_{22}(1-d_{f}\nu_{13}\nu_{31}) & d_{m}E_{22}(\nu_{32}+d_{f}\nu_{12}\nu_{31}) & & & \\ & & E_{33}(1-d_{f}d_{m}\nu_{12}\nu_{21}) & & & \\ & & & \Delta d_{m}d_{f} & & \\ & & & & \Delta d_{m}d_{f} & \\ & & & & & \Delta d_{m}d_{f} \end{array}\right]$$

where $d_{f}$ and $d_{m}$ is introduced as the damage variables for fiber and matrix, expressed as (5). Δ is given by Equation (6) as follows:

(5) $$d_{f}=(1-d_{ft})(1-d_{fc}),\;d_{m}=(1-d_{mt})(1-d_{mc})$$

(6) $$\Delta =1-d_{f}d_{m}\nu_{12}\nu_{21}-d_{m}\nu_{23}\nu_{32}-d_{f}\nu_{13}\nu_{31}-2d_{f}d_{m}\nu_{21}\nu_{32}\nu_{13}$$

Research has confirmed that strain rate positively affects the strength of CFRP composite material. A logarithmic model for describing the effect of strain rate hardening on the strength of fiber-reinforced composites was proposed in refs. [43,44]. To avoid abnormal material failure in the initial low strain rate deformation stage, Xu et al. [45] supplemented an exponential strength expression for the low strain rate range, as shown in Equation (7):

(7) $$S^{ij}\left(\dot{\varepsilon }\right)=\left\{\begin{array}{cc} S_{0}^{ij}\left(1+C_{1}{\dot{\varepsilon }}^{C_{2}}\right) & \dot{\varepsilon }<{\dot{\varepsilon }}_{C}\\ S_{0}^{ij}\left(1+C_{3}ln\frac{\dot{\varepsilon }}{\dot{\varepsilon_{0}}}\right) & \dot{\varepsilon }\geq {\dot{\varepsilon }}_{C} \end{array}\right.$$

where $\dot{\varepsilon }$ and $S^{ij}\left(\dot{\varepsilon }\right)$ are strain rate and corresponding strength. $S_{0}^{ij}\;(ij=1t,\;1c,\;2t,\;2c,\;12,\;13,\;23)$ denotes quasi-static strength. $\dot{\varepsilon_{0}}$ is the reference strain rate. ${\dot{\varepsilon }}_{C}$ is the critical strain rate. $C_{1}$, $C_{2}$, and $C_{3}$ are three material constants. Table 3 lists the values of the parameters related to the strain rate effect, referring to [45].

**Figure 4:**
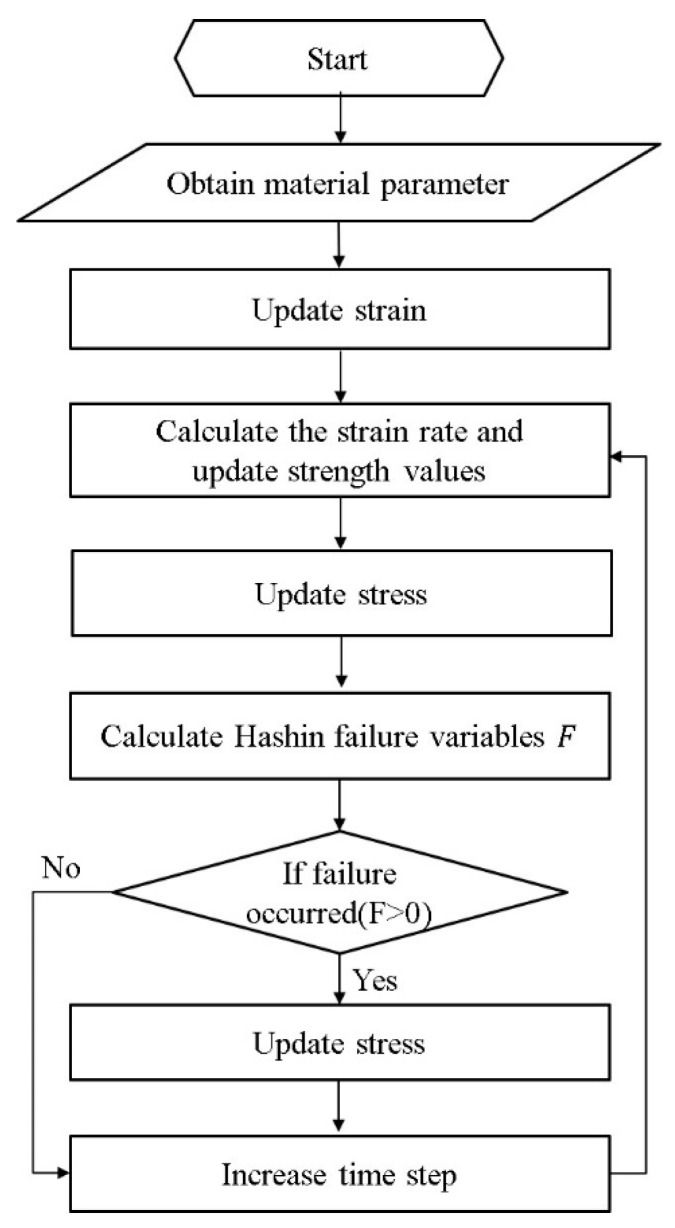
Flowchart of VUMAT for CFRP laminates.

ABAQUS/Explicit provides users with a customizable user material subroutine interface (VUMAT). The above material damage model and strain rate effect are implemented in VUMAT. The calculation procedure is shown in Figure 4.

#### 2.2.2. Inter-Laminar Damage Model

**Figure 5:**
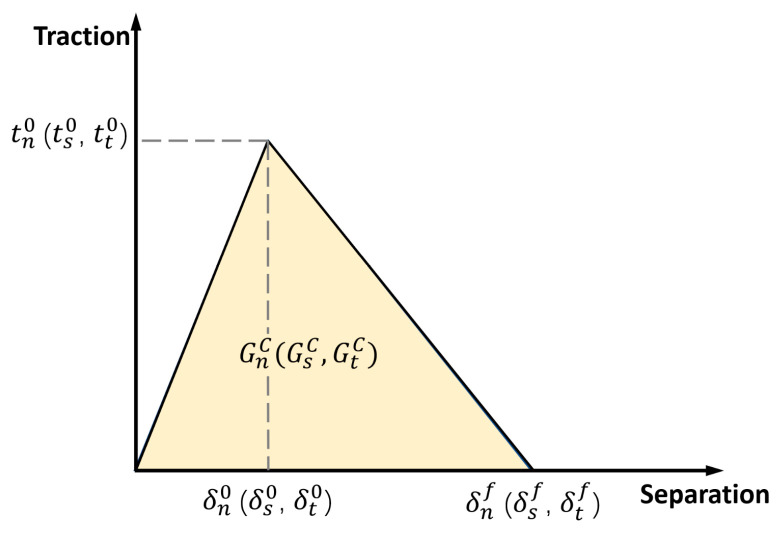
The bilinear traction—separation response of CZM.

The cohesive zone model (CZM) is an efficient way to simulate the delamination of laminates. The damage and failure are defined in terms of traction—separation behavior. A typical bilinear constitutive and progressive damage traction—separation response is shown in Figure 5. When the relative displacement of separation reaches $\delta_{i}^{0}$, traction stress increases to $t_{i}^{0}$, representing the peak value of nominal stress; hereafter, cohesive damage is initiated. $\delta_{i}^{f}$ is the failure value of relative displacement. The subscript i=n, s, t denotes the purely normal direction and two in-plane shear directions.

The damage initiation is determined using mixed-mode criteria for the three purely directional damage modes. Equation (8) is the quadratic nominal stress criterion expression. Damage initiates when a quadratic interaction function involving the nominal stress ratios reaches a value of one:

(8) $${\left\{\frac{\langle t_{n}\rangle }{t_{n}^{0}}\right\}}^{2}+{\left\{\frac{t_{s}^{0}}{t_{s}^{0}}\right\}}^{2}+{\left\{\frac{t_{t}^{0}}{t_{t}^{0}}\right\}}^{2}=1$$

The symbol ⟨⟩ is the Macaulay bracket with an expression of $\left\langle x\right\rangle =(x+\left|x\right|)/2$), signifying that damage would not initiate under pure compressive deformation.

Damage evolution is defined based on the dissipated energy by damage, which is numerically equal to the area bounded by the traction—separation curve and the abscissa axis (Figure 5). The damage evolution, based on the Benzeggagh—Kenane fracture criterion, is given by;

(9) $$G_{n}^{C}+\left(G_{s}^{C}-G_{n}^{C}\right){\left\{\frac{G_{s}+G_{t}}{G_{n}+G_{s}+G_{t}}\right\}}^{\eta }=G^{C}$$

where $G_{n}^{C}$ and $G_{s}^{C}$ refer to the critical fracture energies required to cause failure in the normal and shear directions, respectively. η is a material parameter related to the mixed mode, with a value of 2 [46]. Table 4 shows the parameters of the cohesive elements determined based on the intralaminar material properties as well as ref. [46].

#### 2.2.3. Details of the Finite Element Model

**Figure 6:**
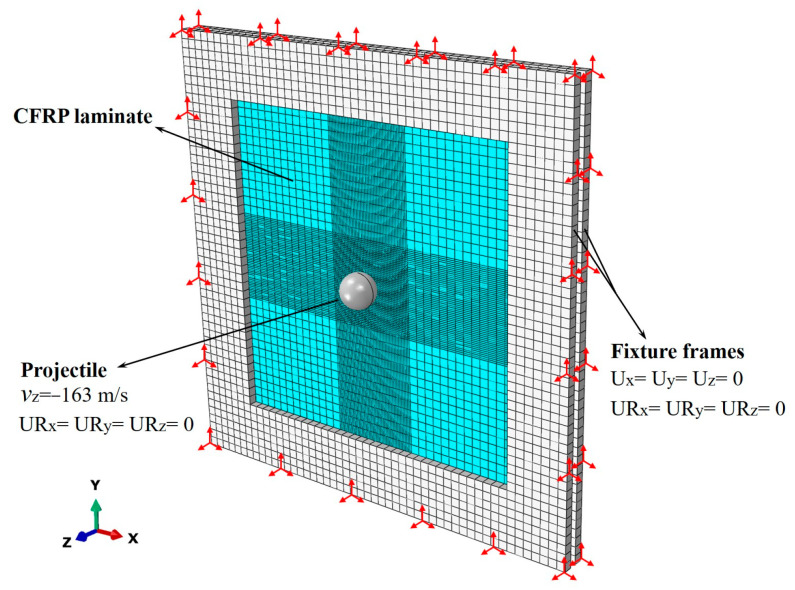
FE model of HVI for the CFRP laminate.

An assembly, including projectiles, CFRP laminates, and fixture frames, is built in ABAQUS CAE software (version 6.13-4), as shown in Figure 6. The stainless-steel spherical projectile is modeled as a rigid body, defining a mass of 33 g to the reference point at the centroid. A pair of fixture frames with 180 mm × 180 mm cut-outs are meshed by 8-node reduced integration solid elements (C3D8R) with a density of 7.89 g/cm^3^, an elastic modulus of 210 GPa, and a Poisson’s ratio of 0.3. They are placed on both sides of the laminate.

**Figure 7:**
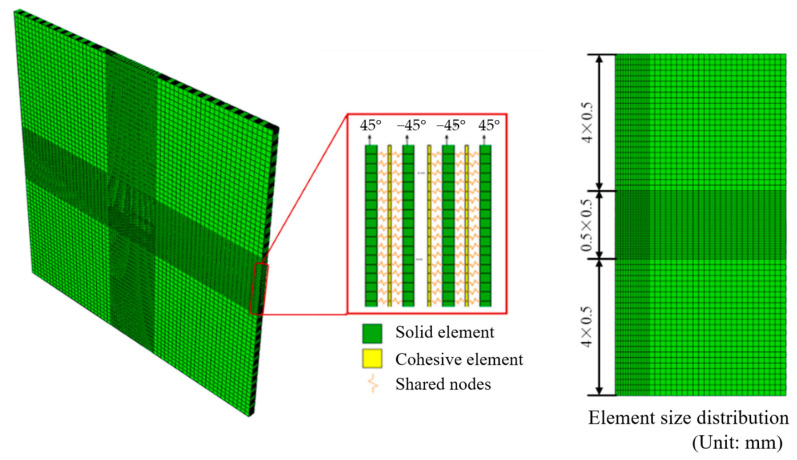
Element types and meshes of the CFRP laminate.

The CFRP laminate model in Figure 7 is established layer by layer employing C3D8R solid elements. Each layer in thickness represents an actual ply of the CFRP laminate. Additionally, zero-thickness 8-node three-dimensional cohesive elements were inserted between adjacent layers to realize delamination failure. The cohesive elements share nodes with elements in adjacent layers. There are a total of 55 elements through the thickness of the laminate, including 28 ply elements and 27 cohesive elements. Convergence validation of mesh sizes is carried out to balance the calculation efficiency and accuracy and determine the meshing scheme for the finite element model. The meshes of the central area 50 mm × 50 mm of the laminate model are refined to a size of 0.5 mm × 0.5 mm. The remaining elements are 4 mm in size. The model consists of 161728 C3D8R elements and 155952 COH3D8 elements.

General contacts are set between the contact surfaces of the projectile, the laminate, the clamp frame, the laminate, and the laminate self, defining the tangential behavior with penalty friction formulation and “Hard” contact for normal behavior. The coefficient of friction is set to 0.3. The fixture frames are fully constrained. The projectile’s rotational degrees of freedom are constrained, and an initial velocity of 163 m/s perpendicular to the laminate is defined for the projectile. The boundary conditions of the model are shown in Figure 6.

## 3. Results

### 3.1. Velocity History and Energy

**Figure 8:**
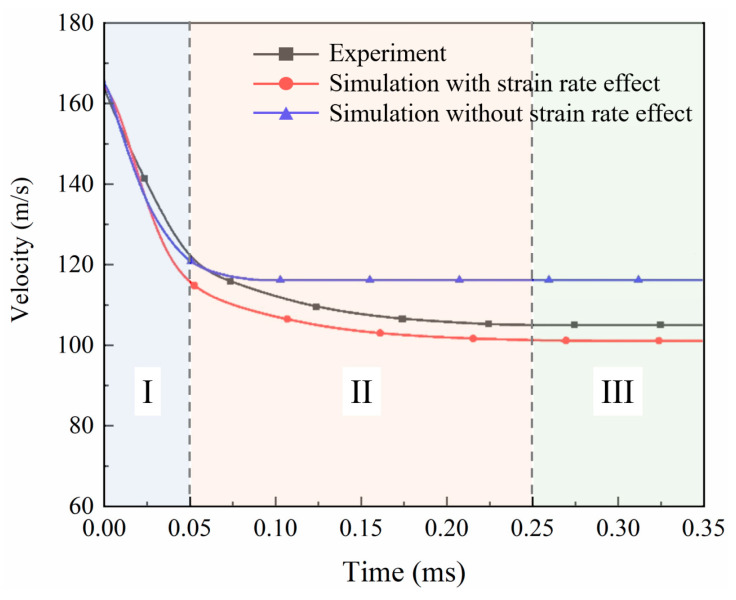
The velocity history curves from the experiment and simulations.

Three HVI tests are carried out under the same conditions, and the impact speed of the projectile recorded by the velocimeter is 163±2 m/s. High-speed photography captures impact process images at a high frequency, allowing users to obtain the impact velocity history of the projectile. In order to show the role of the material strain rate sensitivity effect in the simulation, two methods were used, one considering the strain rate sensitivity of the material, and the other not considering the strain rate sensitivity of the material. Figure 8 plots the projectile velocity history curves output from both methods and the experimental results.

The projectile velocity history curves from the experiments, represented by the black curves in Figure 8, can be categorized into three stages according to the rate of decrease. In Stage I, the projectile’s velocity drops rapidly after it reaches the surface of the laminate (Stage I: 0–0.05 ms). When the laminate begins to fail under the impact force, the velocity drop rate gradually decreases, and this phase lasts about 0.2 ms (Stage II: 0.05–0.25 ms). Finally, the projectile completely penetrates the laminate, and the velocity tends to be constant (Stage III: 0.25–0.35 ms). The remaining velocity of the projectile leaving the laminate is 102.78 m/s. According to the simulation results, without considering the strain rate effect (represented by the blue curve with triangular symbols), the entire impact process is 0.25 ms, significantly shorter than the actual time, and the velocity change trend is also different. When considering the strain rate effect (represented by the red curve with circular symbols), the simulation results are highly similar to the experimental results, except that the projectile velocity is slightly lower in the first phase.

The residual kinetic energy is obtained through residual velocity. Kinetic energy loss is calculated as the difference between initial kinetic energy and residual kinetic energy. Table 5 lists the remaining velocity and kinetic energy loss and corresponding errors between the experiment and simulations. The addition of the strain rate effect in the material properties of the laminate significantly reduces the error of residual velocity by 5.6% and kinetic energy loss by 8.19%. The above results verify the effectiveness of the simulation model, and further research is carried out based on this model.

### 3.2. Damage Profiles

**Figure 9:**
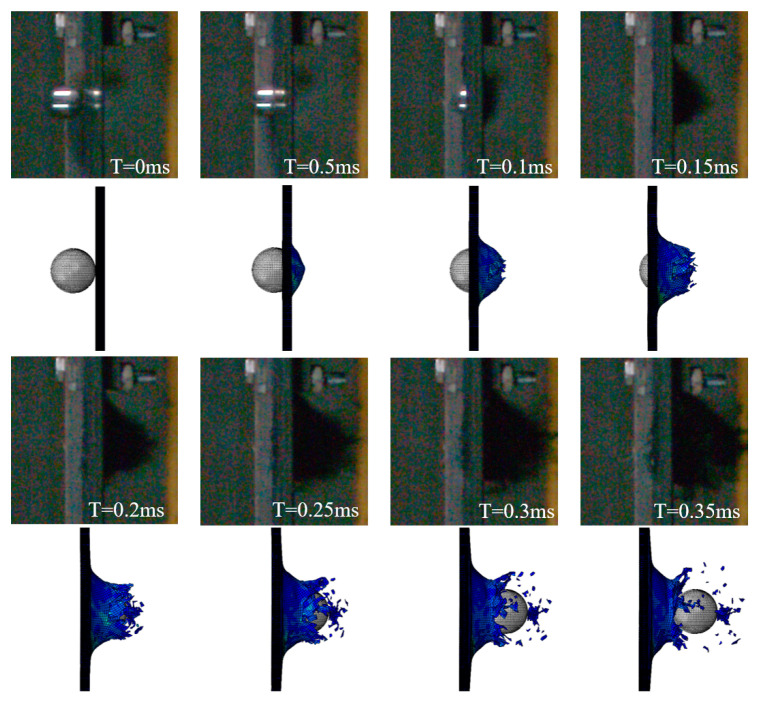
The HVI process of photography and simulation considering the strain rate effect.

**Figure 10:**
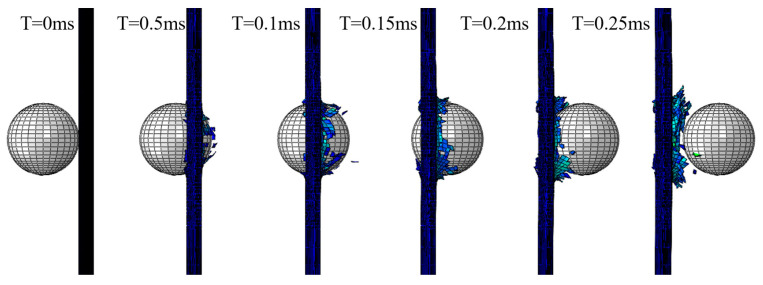
The HVI damage process of the simulation without considering the strain rate effect.

**Figure 11:**
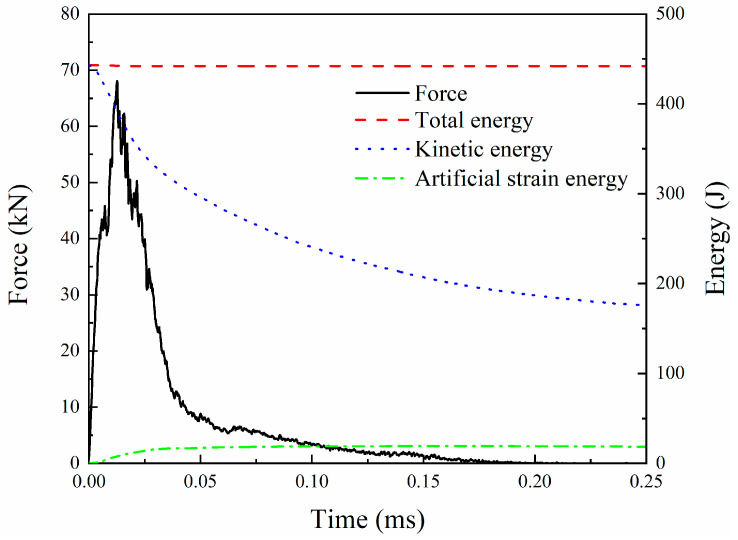
Impact force and energy versus time curve of the finite element model.

From the comparison of the images showing the damage and failure of the laminate, there is a pronounced conical bulge in the impacted area (as seen in Figure 9). A similar phenomenon is not seen in the simulation results, as shown in Figure 10. Compared with the simulation without considering the strain rate effect, the impact process is more realistically reproduced by considering the strain rate effect. The impact force and energy histories are plotted in Figure 11. The impact force rises rapidly before 0.02 ms. With the damage to the laminate, the impact force first decreases rapidly and then tends to zero slowly. The projectile kinetic energy decreases from 422.88 J to 165.82 J. The artificial strain energy is used by the computer software to control hourglass deformation in the elements. In other words, high levels of artificial strain energy can result in possible significant errors and should, therefore, be as low as possible. The artificial strain energy output from the simulation in this study was kept below the level of 20 J, which is less than 5% of the total energy, indicating that the simulation has a small error caused by the artificial strain energy.

**Figure 12:**
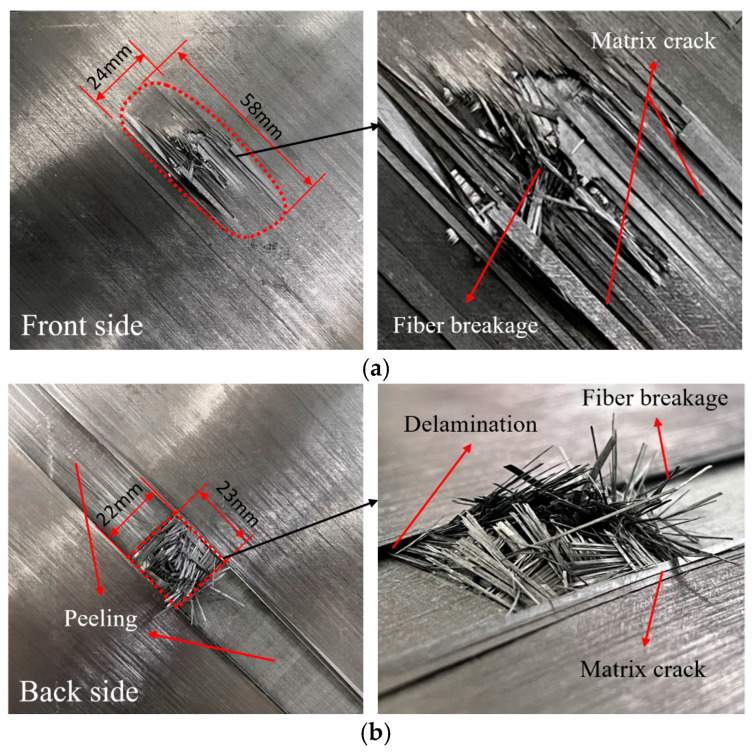
Damage from the (**a**) front and (**b**) back views of the CFRP laminate.

The local failure of the sample after the HVI is shown in Figure 12. Both the front side and backside of the laminate are plotted. From the front side of the laminate (Figure 12a), a damaged area with a size of 22 mm × 58 mm is observed, with the length along the longitudinal direction. There are fiber failures at the impact center and matrix cracks around it. A combination of broken fibers, matrix cracking, and delamination forms raised slender fiber tows. Viewed from the back of the laminate (Figure 12b), a visible 22 mm × 23 mm square failure area is formed after the penetration by the projectile, and a large area of peeling of the surface layer was observed. From the enlarged side view on the right of Figure 12b, it can be seen that there is a large number of broken fibers in the penetration area accompanied by matrix cracking, and delamination is observed next to the surface peeling area.

**Figure 13:**
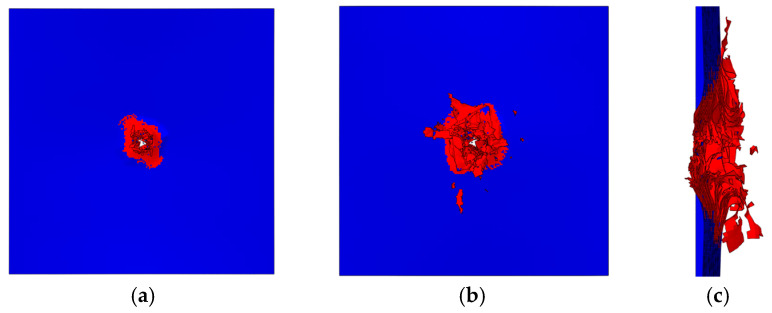
The simulation result of penetration morphology after impact: (**a**) front side, (**b**) back side, and (**c**) side sectional view.

The whole process of HVI is reproduced by simulation (seen in Figure 9), and the penetration morphology of the laminate is shown in Figure 13. Matrix cracks are indicated in red, and blue indicates no intra-laminar damage. The impact created a hole in the center of the laminate that was slightly larger than the projectile. Elements with fiber tensile failure are removed, forming an impact hole, and there is a large amount of matrix damage around the hole. From the side sectional view (Figure 13c), the failure in the layer caused some elements to fly out as fragments. In addition to intra-layer failures, delamination can also be observed.

**Figure 14:**
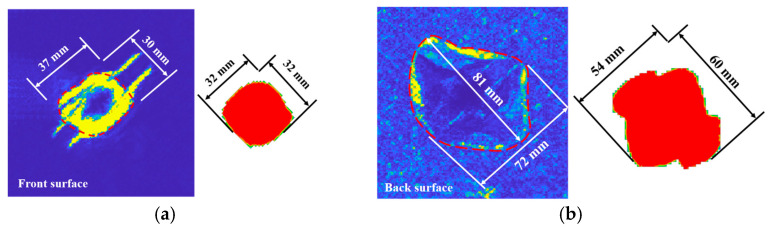
The surface delamination profile of the specimen after impact, (**a**) front surface, and (**b**) back surface.

**Figure 15:**
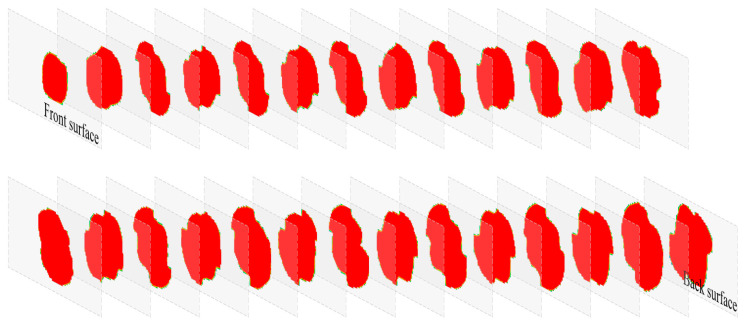
Delaminated areas from the front surface to the back surface.

In order to verify the validity of the simulated delamination damage, an ultrasonic C-scan technique was used to plot the delamination contours of the front and back surfaces of the impacted specimen, as shown in Figure 14. The simulation could output the delamination area of each pair of adjacent layers of the laminate. A comparison of the surface delamination contours in Figure 14 shows that the contours and dimensions of the simulated delamination areas (the red pattern on the right) are close to those of the test. In addition, all the delaminated areas, composed of failed cohesive elements from the front surface to the back, are drawn in Figure 15. The delaminated areas are embedded in grey translucent rectangular base plates measuring 100 mm × 50 mm. The delaminated area increases from the front to the back. The total delaminated area is 35,109 mm^2^.

## 4. Discussion

The above experimentally verified finite element model provides us with convenience for discussing the influence of different factors on the high-speed impact penetration performance of laminates. By changing the projectile geometry or simulation conditions, we can discuss the effect of impact velocity, impactor shape, and target oblique angle on the HVI response of CFRP laminates.

### 4.1. Effect of Impact Velocity

**Figure 16:**
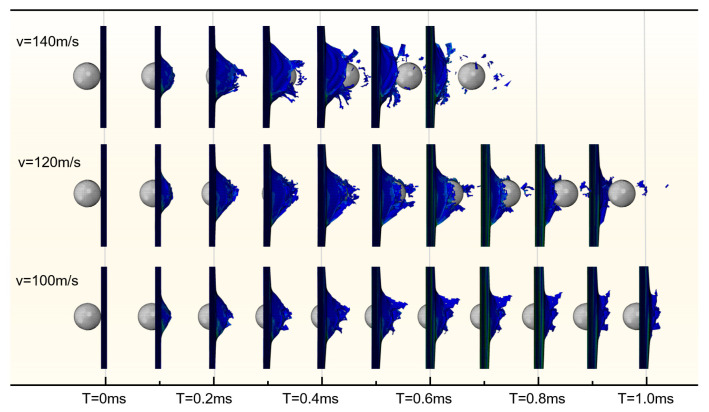
HVI process under different impact velocities.

To study the impact velocity’s effect on the laminate’s perforation performance, simulations were performed at four different impact velocities: 163 m/s, 140 m/s, 120 m/s, and 100 m/s. The HVI process under different impact velocities is plotted in Figure 16. The results show that the laminate target cannot resist penetration by projectiles with a velocity of 120 m/s or higher. The penetration time increases as the projectile velocity decreases. The impact with a projectile velocity of 100 m/s does not entirely penetrate the laminate, although each layer has some degree of damage. The projectile stops moving forward and starts to rebound at about 0.4 ms, indicating that the velocity of 100 m/s is less than the ballistic limit of the laminate.

**Figure 17:**
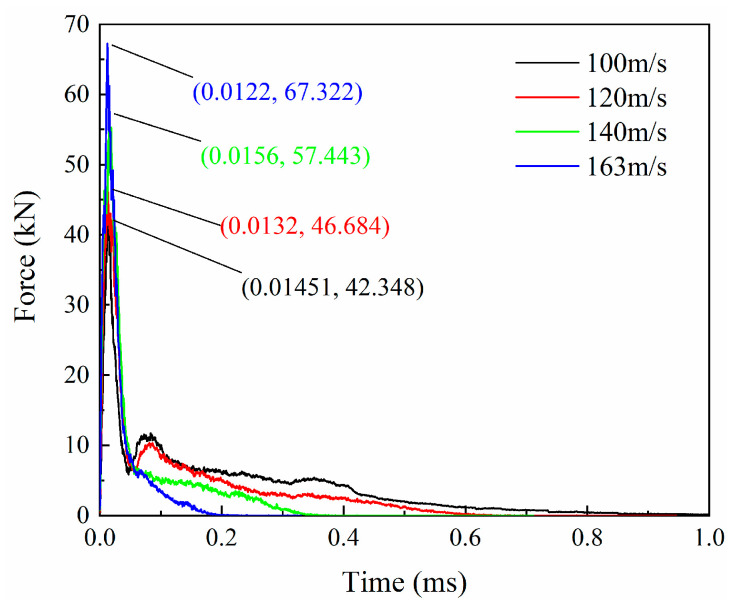
Force histories under different impact velocities.

**Figure 18:**
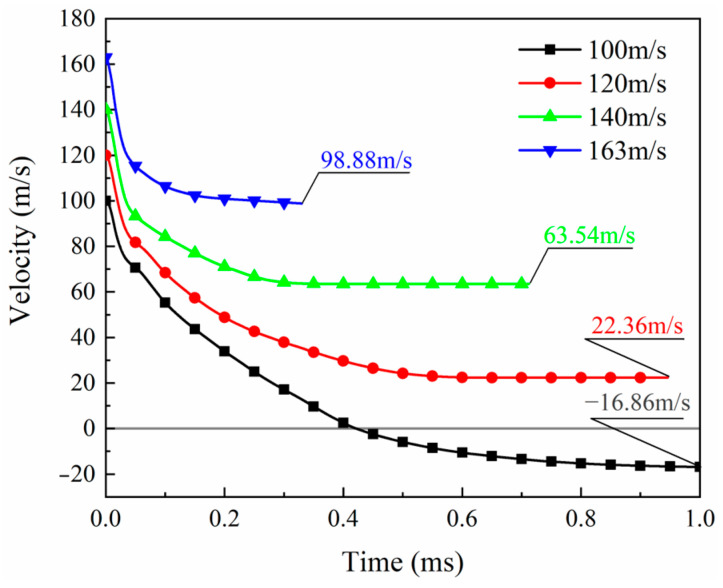
Velocity histories under different impact velocities.

**Figure 19:**
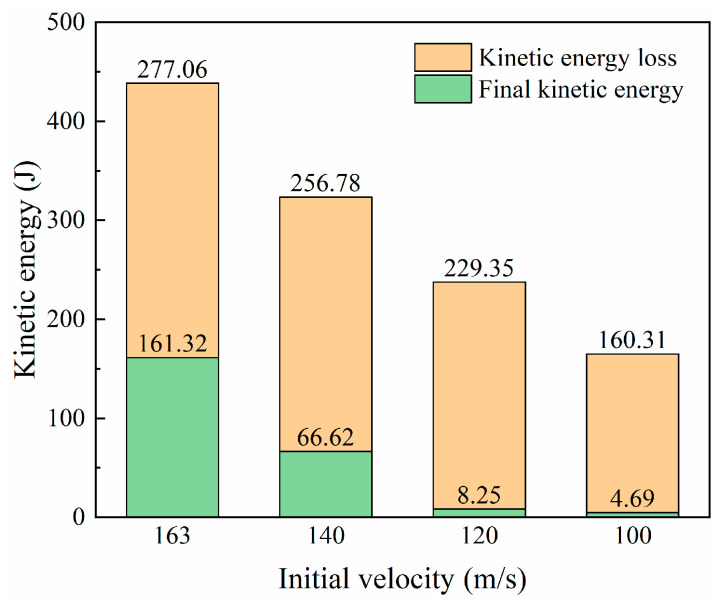
Final kinetic energy and kinetic energy loss of projectile with different initial velocities.

The contact force during the impact process can be calculated from the projectile acceleration history output by the simulation, and the force history curves under different impact velocities are plotted in Figure 17. After the projectile contacts the laminate, the force rapidly increases to a peak and then decreases sharply. High velocities result in high impact peak forces. Low impact velocities (100 m/s and 120 m/s) have a second peak force of around 10 kN at about 0.08 ms. According to the analysis of the impact process, the relatively low impact energy cannot directly penetrate the laminate, and the blockage of the latter plies leads to the appearance of the second peak. When the impact energy is high enough, this phenomenon of staged penetration does not exist. For higher velocities (140 m/s and 163 m/s), the impact force gradually drops to zero after the first peak. Comparing the velocity history curves of different impact velocities in Figure 18, it is found that a high initial impact velocity leads to a high remaining velocity. Figure 19 shows the change in kinetic energy of the projectile calculated from the change in velocity. As the projectile’s initial velocity decreases, the final kinetic energy decreases, and the kinetic energy is lost.

**Figure 20:**
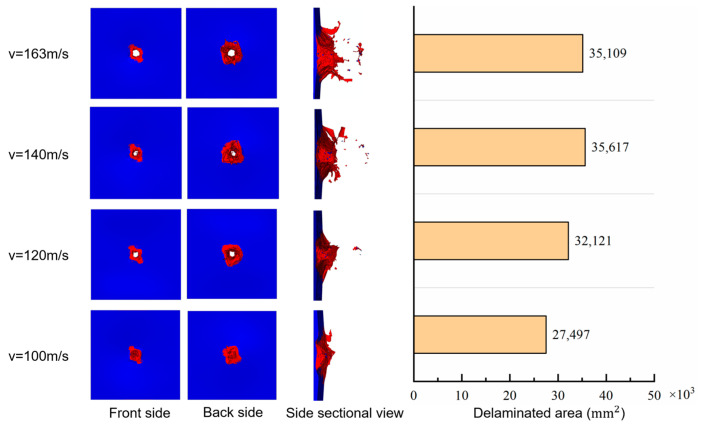
Damages of laminates impacted under different velocities.

The laminate damage after impact is shown in Figure 20. Impacts above the ballistic limit all pierce a hole in the target’s center, close to the projectile’s size. The matrix cracked area surrounding the hole is similar in shape and size, while the crack area of the matrix under the impact of 100 m/s velocity is relatively small. The total delamination area increases with increasing velocity in the impact velocity range from 100 m/s to 140 m/s, and decreases slightly when the velocity reaches 160 m/s. This indicates that the delamination area may reach a limit value under high-speed impact above 140 m/s, while below 140 m/s, the velocity positively correlates with the delaminated area.

### 4.2. Effect of Impactor Shape

**Figure 21:**
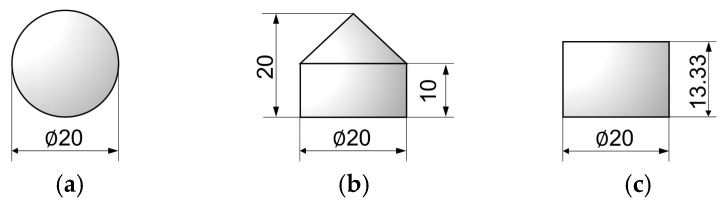
Geometries of the three impactors: (**a**) sphere, (**b**) cone, and (**c**) cylinder. (Unit: mm).

Considering that real-world impactors may have different geometries, this study considered two other impactors with different shapes, namely a cone (Figure 21b) and a cylinder (Figure 21c). The conical-head impactor represents foreign objects with sharp protrusions. The contact between the cylindrical impactor and the target is flat-to-flat, representing blunt foreign objects. The three impactors’ geometric design ensures they have the same projection in the impact direction and volume.

**Figure 22:**
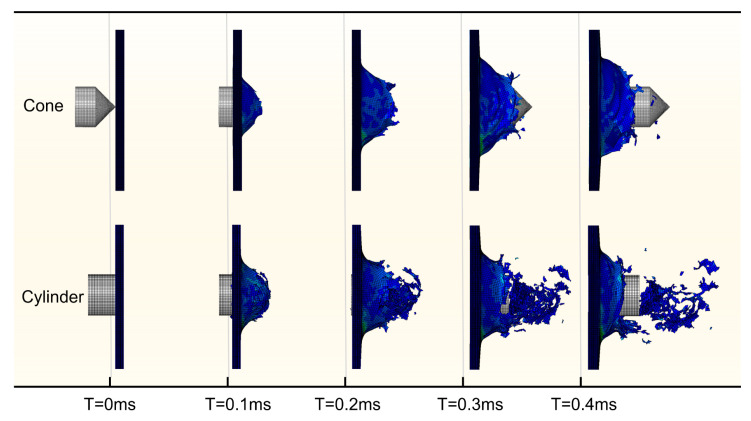
HVI damage process for conical and cylindrical projectiles.

From the perspective of the impact damage process plotted in Figure 22, when the laminate is impacted by a cone-shaped object, a small initial penetrated area is formed by compression of the cone tip and then gradually expands with tensile stress until the projectile passes through. After the end face of the cylindrical projectile comes into contact with the surface of the target, the deformation of the laminate leads to the edge of the end face and the laminate forming an annular contact area. The contact area first fails under the combination of compression and shear in a short period, and then the elements close to or in the back surface fail under tensile stress. Shear effects at the edge of the cylindrical end face produce typical plug failures with many cell fragmentation separations.

**Figure 23:**
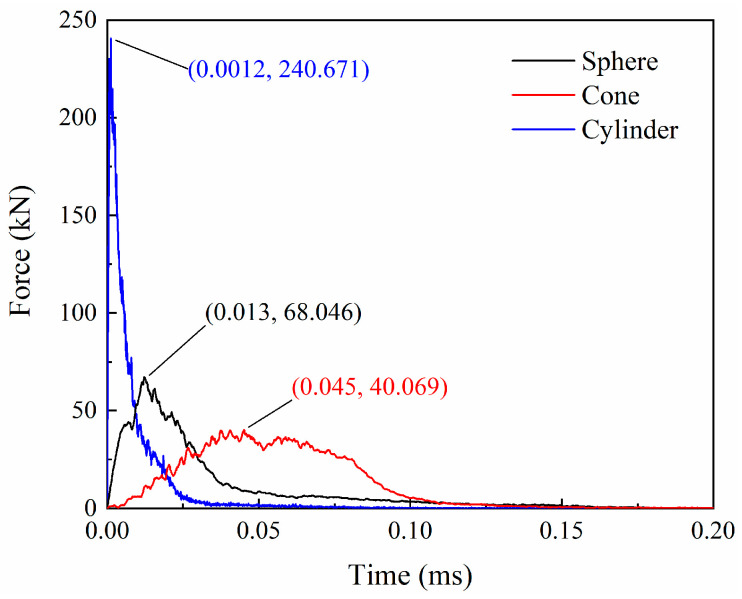
Force histories for different projectiles.

**Figure 24:**
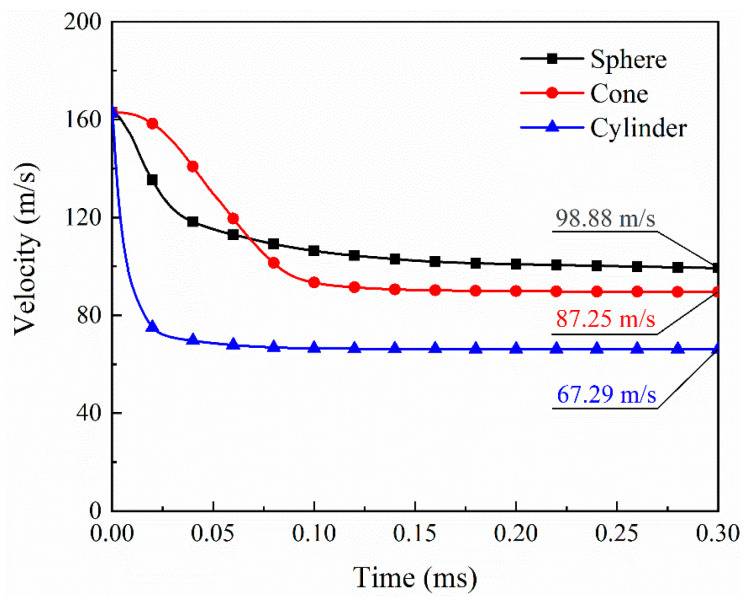
Velocity histories for different projectiles.

**Figure 25:**
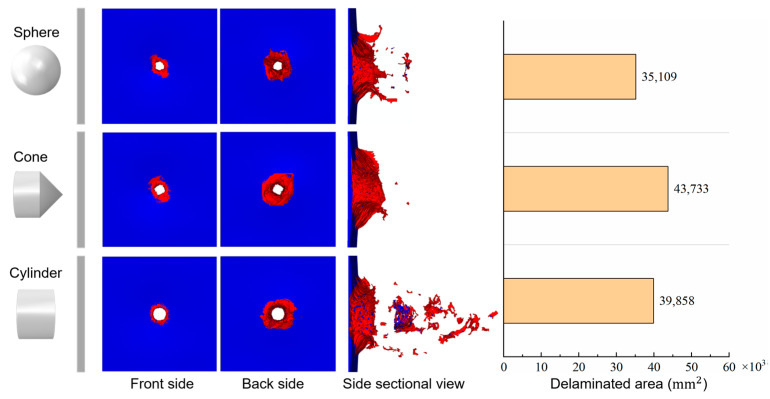
Damages of laminates impacted by different projectiles.

Figure 23 and Figure 24 give the force and velocity histories for the different projectiles, respectively. Compared with the impact of spherical projectiles, the impact process of conical projectiles lasts longer, and the impact resistance is lower, with a peak force of 40.07 kN. Conversely, the cylindrical projectile has a shorter impact time and a much higher peak impact force of 240.67 kN. In the initial stage of the impact, the velocity of the conical projectile decreases slowly due to the relatively slow increase in resistance, and the final velocity is 87.25 m/s. The cylindrical projectile has the fastest drop in velocity, with a final velocity of 67.29 m/s. The remaining velocities of the three projectiles indicate that laminates absorb the most energy for blunt foreign objects in HVI. When the impact object is conical, the energy absorption is greater than that of a spherical projectile. This is not the same as the effect of the projectile shape on the impact peak force. Damages of laminates under different projectile impacts are given in Figure 25. Three projectiles penetrated the laminate to form holes of a similar size. The shear effect of the cylindrical projectile impact makes the edges of the hole more regular. The conical projectile impact caused extensive matrix cracking, and extensive tearing was observed in the back surface layer. The delaminated area of the laminate under the impact of the conical projectile is the largest, followed by the cylindrical projectile, and the smallest by the spherical projectile, as shown in Figure 25.

### 4.3. Effect of Oblique Angle

**Figure 26:**
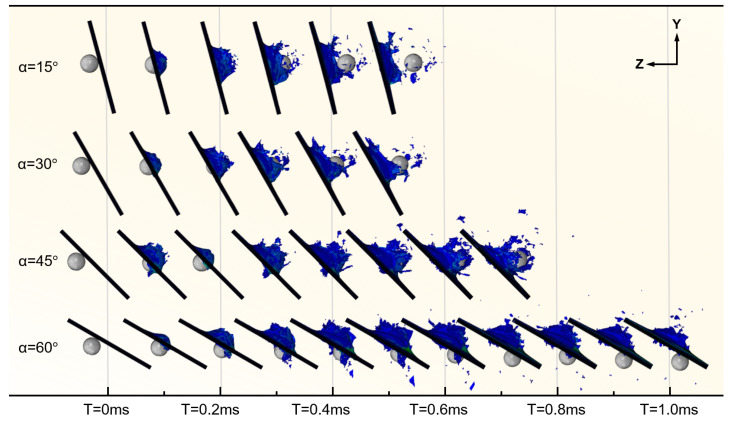
HVI process simulation for CFRP laminates with different oblique angles.

This subsection investigates the effect of different target oblique angles on the HVI response. The angle between the plate and the vertical plane, α, ranges from 0° (normal incidence) to 60°. Figure 26 indicates that when the inclination angle is not greater than 45°, as the inclination angle of the laminate increases, the path length of the projectile penetrating the laminate increases, and the penetration time increases accordingly. However, when the inclination angle reached 60°, the projectile failed to pass through the laminate to the other side. It is evident that a larger oblique angle results in a larger area of damage.

**Figure 27:**
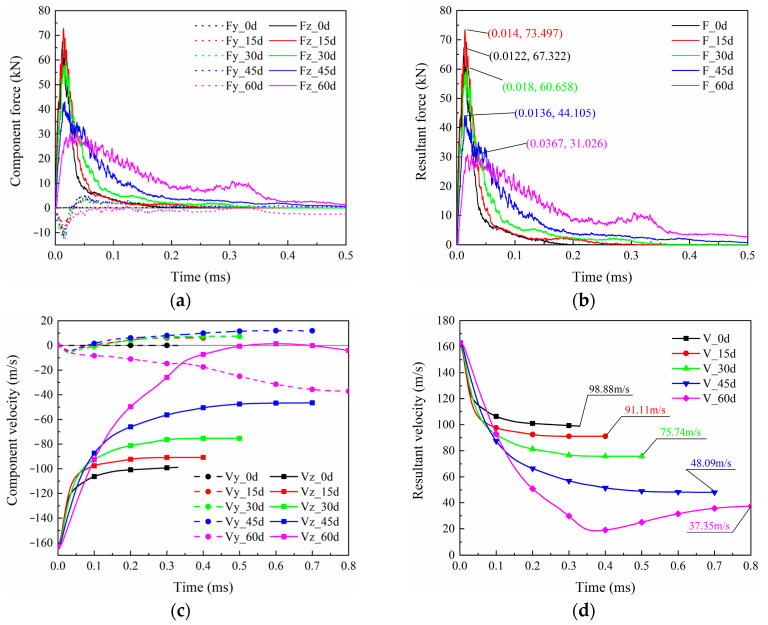
HVI history curves for (**a**) component force, (**b**) resultant force, (**c**) component velocity, and (**d**) resultant velocity.

**Figure 28:**
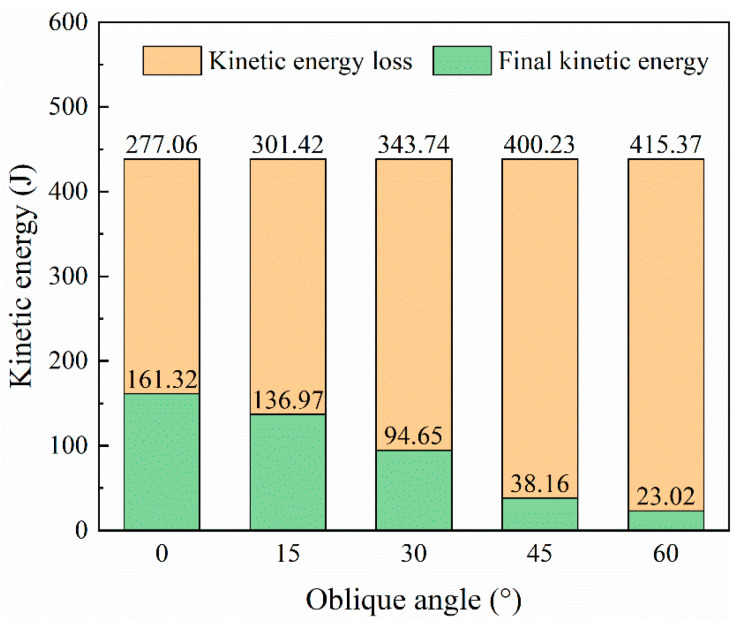
Final kinetic energy and kinetic energy loss of projectiles for different oblique angles.

During the impact process, after the projectile contacts the oblique target, in addition to moving in the direction of initial velocity, it also moves in the vertical direction due to the vertical force. The dashed and solid lines in Figure 27a represent the horizontal (z) and vertical (y) component forces of the projectile, respectively. The vertical force is significantly smaller than the horizontal force. The horizontal force is significantly larger than the vertical force, which results in resultant force curves (Figure 27b) that are almost identical to the horizontal force curves. Generally, each component has a trend of rising first and then falling. For the 60° case, during the long-term contact with the projectile laminate, the rebound of the laminate caused another peak in the horizontal force in the time interval of 0.25–0.35 ms. Another conclusion is that larger oblique angles lead to smaller peak forces. The case with a 15° oblique is an exception, and the horizontal force is slightly higher than that of the 0° case. Except for the 60° case, as the laminate is damaged, the direction of the vertical force changes from downward to upward and continues until the end of penetration. The velocity history curves in Figure 27c indicate that a large inclination angle makes the projectile velocity drop slower, and more kinetic energy can be absorbed due to the larger damaged area. The residual velocities of the case with oblique angles from 15° to 60° are 91.11 m/s, 75.74 m/s, 48.09 m/s, and 37.35 m/s, respectively, as seen in Figure 27d. Correspondingly, the larger the oblique angle, the more kinetic energy the projectile loses (Figure 28). In other words, the increase in the oblique angle is beneficial to lifting the ballistic limit of the laminate.

**Figure 29:**
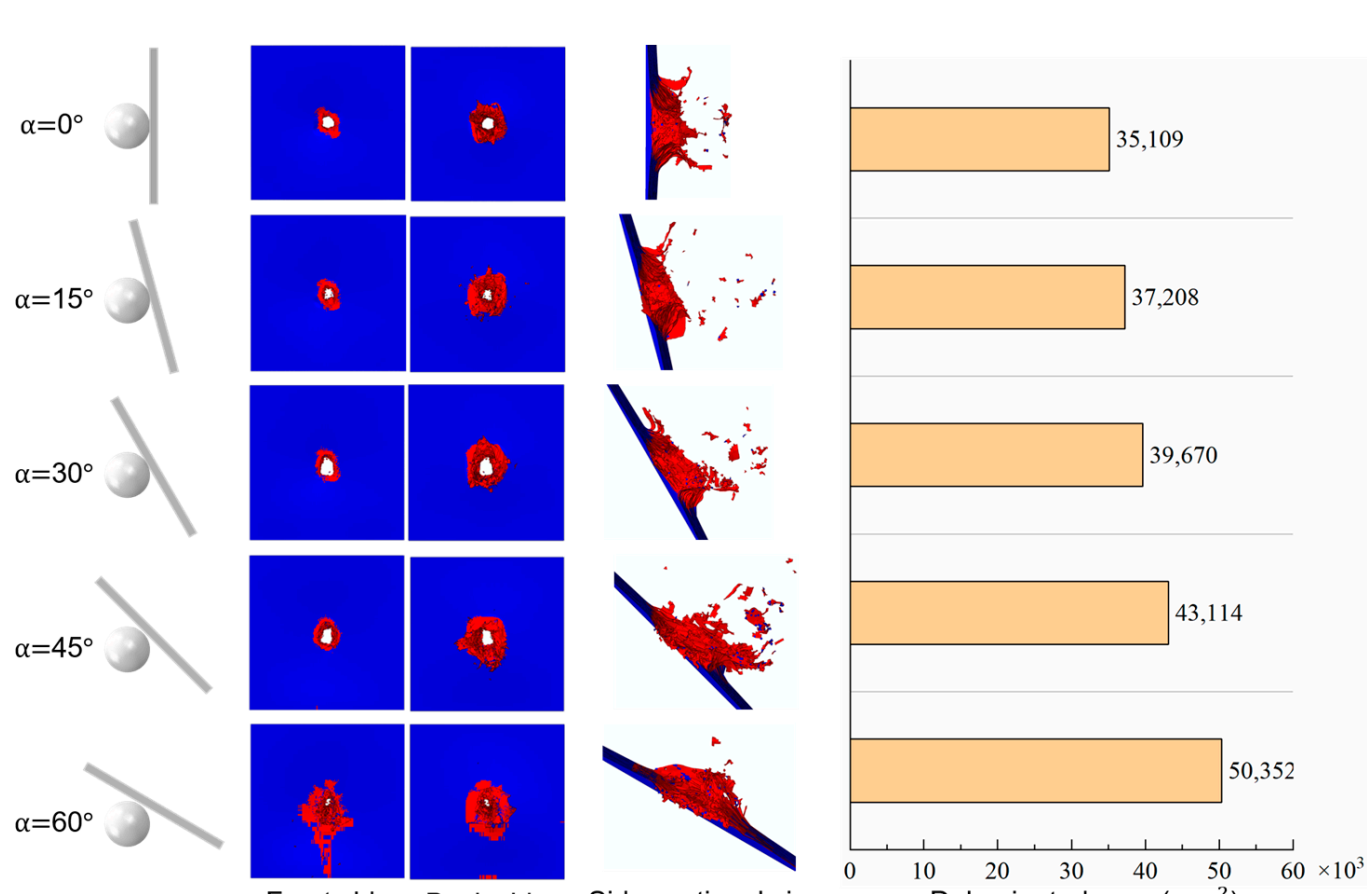
HVI damages of laminates with different oblique angles.

Comparing the damage of laminates with different oblique angles in Figure 29, the larger the oblique angle, the larger the penetration hole (if there is penetration), and the larger the damaged area of the matrix. For the largest oblique angle impact cases (α=60°), the matrix cracks extended to the laminate’s edge. The total delaminated area is also positively correlated with the oblique angle.

## 5. Conclusions

In this work, high-velocity impact experiments and finite element simulations are performed to investigate the perforation performance of CFRP laminates for rail vehicles. The projectile penetrates the laminate, and failure modes include fiber breakage, matrix cracking, and delamination. The projectile velocity history is obtained from the image analysis of the impact process recorded by the high-speed camera in the test, and the consistency with the simulation results verifies the validity of the model. Based on this baseline model, we study the effects of impact velocity, projectile shape, and target inclination on the perforation resistance of laminates. According to the simulation results, the following conclusions are extracted:

- The ballistic limit of the laminate is within the speed range of 100–120 m/s. There is a positive correlation between impact velocity and peak impact force, impact velocity, and projectile kinetic energy loss. The delaminated area decreased by 22.8% when the speed decreased from 140 m/s to 100 m/s. In contrast, the delamination area at an impact velocity of 163 m/s differs from that at 140 m/s by only 1.4%.
- The shear plugging by the cylindrical projectile’s flat end produces many material fragments in the central area. The peak impact force is highest for the cylindrical projectile, 253.7% higher than in the spherical projectile, and 500.6% higher than in the case of the conical projectile. However, the residual velocity of the spherical projectile is higher than that of the other two projectiles, 13.3% higher than that of the conical projectile, and 46.9% higher than that of the cylindrical projectile. The conical projectile penetrated the laminate with the most negligible impedance but produced the largest delaminated area compared to the other two projectiles.
- The oblique angle of the target significantly influences the perforation performance of the laminate. Increasing the oblique angle can reduce the peak impact force. The longer the impact path of the projectile, the larger the damaged area of the laminate, which is conducive to the consumption of the impact energy of the projectile. Compared with the baseline model, when the oblique angle of the laminate is 60°, the peak impact force decreases by 57.8%, the residual velocity decreases by 62.2%, and the delaminated area increases by 43.4%.
- The results obtained in this study show that CFRP laminates currently used in rail vehicles cannot resist the risk of high-speed impacts of foreign objects that may be faced during operation. Optimization of the layup and biomimetic sandwich structure can be used to improve the structural impact resistance in practical applications. In addition, if the train encounters hailstorms, windstorms, and other weather conditions during operation, it is necessary to slow down or stop the operation as appropriate.

## Figures and Tables

**Table 1:** Expressions of 3D-Hashin criterion.

Damage Modes	Damage Criteria	Degradation Constant
Fiber tensile $\left(\sigma_{11}\geq 0\right)$	$F_{f}^{t}={\left(\frac{\sigma_{11}}{S_{1t}}\right)}^{2}+\frac{\tau_{12}^{2}+\tau_{13}^{2}}{S_{12}^{2}}\geq 1$	$d_{ft}=0.07$
Fiber compression ($\sigma_{11}\leq 0$)	$F_{f}^{c}={\left(\frac{\sigma_{11}}{S_{1c}}\right)}^{2}\geq 1$	$d_{fc}=0.14$
Matrix tensile $\left(\sigma_{22}+\sigma_{33}\geq 0\right)$	${F_{m}^{t}=\left(\frac{\sigma_{22}+\sigma_{33}}{S_{2t}}\right)}^{2}+\left(\frac{\tau_{23}^{2}-\sigma_{22}\sigma_{33}}{S_{23}^{2}}\right)+\frac{\tau_{12}^{2}+\tau_{13}^{2}}{S_{12}^{2}}\geq 1$	$d_{mt}=0.2$
Matrix compression $(\sigma_{22}+\sigma_{33}\leq 0$)	${F_{m}^{c}=\left(\frac{\sigma_{22}+\sigma_{33}}{2S_{23}}\right)}^{2}+\left[{\left(\frac{Y_{c}}{{2S}_{23}}\right)}^{2}-1\right]∙\frac{\sigma_{22}+\sigma_{33}}{S_{2c}}+\left(\frac{\tau_{23}^{2}-\sigma_{22}\sigma_{33}}{S_{23}^{2}}\right)+\frac{\tau_{12}^{2}+\tau_{13}^{2}}{S_{12}^{2}}\geq 1$	$d_{mc}=0.4$

**Table 2:** Material properties of the adherends.

Item	Symbol and Value
Density	$\rho =1.68\;g/{\mathrm{cm}}^{3}$
Poisson’s ratio	$\nu_{12}=\nu_{31}=0.28$, $\nu_{23}=0.35$
Modulus	$E_{11}=157.0\;\mathrm{GPa}$, $E_{22}=E_{33}=7.73\;\mathrm{GPa}$
Shear modulus	$G_{12}=G_{31}=6.20\;\mathrm{GPa}$, $G_{23}=4.60\;\mathrm{GPa}$
Longitudinal strength	$S_{1t}=2355\;\mathrm{MPa}$, $S_{1c}=790\;\mathrm{MPa}$
Transversal strength	$S_{2t}=71\;\mathrm{MPa}$, $S_{2c}=173\;\mathrm{MPa}$
Shear strength	$S_{12}=S_{31}=73.0\;\mathrm{GPa}$, $S_{23}=50\;\mathrm{GPa}$

**Table 3:** The parameters of strain rate effect.

Parameter	$\dot{\varepsilon_{0}}$	${\dot{\varepsilon }}_{C}$	$C_{1}$	$C_{2}$	$C_{3}$
Value	$0.001\;s^{-1}$	$300\;s^{-1}$	0.1	0.123	0.131

**Table 4:** Parameter values of the cohesive elements.

Material Property	Symbol and Value		
Modulus	$E_{nn}=7.73\;\mathrm{GPa}$	$E_{ss}=6.20\;\mathrm{GPa}$	$E_{tt}=6.20\;\mathrm{GPa}$
Strength	$t_{n}^{0}=$ 65.43 MPa	$t_{s}^{0}=$ 98.15 MPa	$t_{t}^{0}=$ 98.15 MPa
Fracture toughness	$G_{n}^{C}=$ 0.37 N/mm	$G_{s}^{C}=3.82$ N/mm	$G_{t}^{C}=3.82$ N/mm
Density	ρ= 1.44 g/cm^3^		

**Table 5:** Comparison of the simulation and experimental results.

	Experiment	Simulation without Strain Rate Effect	Simulation with Strain Rate Effect
Remaining velocity	102.78 m/s	112.52 m/s	98.88 m/s
Error	-	9.48%	3.79%
Kinetic energy loss	264.09 J	229.49 J	277.06 J
Error	-	13.10%	4.91%

## Data Availability

The data presented in this study are available on request from the corresponding author.
